# Efficacy and safety of paritaprevir/ritonavir, ombitasvir, and dasabuvir with ribavirin for the treatment of HCV genotype 1b compensated cirrhosis in patients aged 70 years or older

**DOI:** 10.1097/MD.0000000000009271

**Published:** 2017-12-15

**Authors:** Anca Trifan, Carol Stanciu, Liana Gheorghe, Speranta Iacob, Manuela Curescu, Cristina Cijevschi Prelipcean, Gabriela Stefanescu, Irina Girleanu, Stefan Chiriac, Catalina Mihai, Ciprian Brisc, Adrian Goldis, Ioan Sporea, Egidia Miftode, Simona Bataga, Ion Rogoveanu, Carmen Preda, Florin Alexandru Caruntu, Ana-Maria Singeap

**Affiliations:** a “Grigore T. Popa” University of Medicine and Pharmacy Iasi; b Institute of Gastroenterology and Hepatology, Iasi; c Gastroenterology, “Carol Davila” University of Medicine and Pharmacy; d Gastroenterology and Hepatology Center, Fundeni Clinical Institute, Bucharest; e Infectious Diseases, “Victor Babes” University of Medicine and Pharmacy; f Department of Infectious Diseases, “Victor Babes” Hospital for Infectious and Lung Diseases, Timisoara; g Gastroenterology, Oradea University of Medicine and Pharmacy; h Department of Gastroenterology, County Clinical Hospital, Oradea; i Gastroenterology, “Victor Babes” University of Medicine and Pharmacy; j Department of Gastroenterology and Hepatology Timisoara, The County Hospital Timisoara; k Infectious Diseases, “Grigore T. Popa” University of Medicine and Pharmacy; l “Sf. Parascheva” Infectious Diseases Clinical Hospital, Iasi; m Gastroenterology, Targu Mures University of Medicine and Pharmacy, Targu Mures; n Department of Gastroenterology, Targu Mures Emergency County Hospital; o Internal Medicine, Craiova University of Medicine and Pharmacy; p Internal Medicine Department, Emergency Clinical County Hospital Craiova; q Infectious Diseases, “Carol Davila” University of Medicine and Pharmacy; r “Matei Bals” National Institute for Infectious Diseases, Bucharest, Romania.

**Keywords:** direct-acting antivirals, elderly patients, HCV infection, liver cirrhosis, paritaprevir/ritonavir ombitasvir and dasabuvir

## Abstract

Advanced age has been a major limitation of interferon-based treatment for chronic hepatitis C virus (HCV) infection because of its poor response and tolerability. Direct-acting antiviral (DAA) drug regimens are safe and highly effective, allowing administration of treatment also in elderly. This study aims to assess the efficacy and safety of paritaprevir/ritonavir, ombitasvir, and dasabuvir (PrOD) with ribavirin for the treatment of patients aged ≥70 years with HCV genotype 1b compensated cirrhosis.

A total of 1008 patients with HCV genotype 1b compensated cirrhosis were prospectively treated with PrOD + ribavirin for 12 weeks, between December 2015 and July 2016. Sustained virologic response 12 weeks after the end of treatment (SVR12), adverse effects (AEs), comorbidities, discontinuation, and death rates were recorded. Efficacy and safety of therapy were assessed in patients aged ≥70 years and compared with data from patients <70 years.

There were 117 patients aged ≥70 years, preponderantly females (58.9%), mean age 73.3 ± 2.8 years (range 70–82), and 37 (31.6%) were treatment-experienced. Comorbidities were reported in 60.6% of patients ≥70 years and in 39.8% of those <70 years (*P* < .001). SVR12 rates based on intention-to-treat and per-protocol analyses were 97.4% and 100%, respectively, in patients ≥70 years, compared to 97.8% and 99.6%, respectively, in patients <70 years (*P* = ns and *P* = ns). Severe AEs were reported in 4 (3.4%) patients ≥70 years, compared to 23 (2.6%) in those <70 years (*P* = ns). One death was recorded in a patient aged 79 years (0.9%) and 6 deaths (0.8%) in those <70 years (*P* = ns).

Treatment with PrOD + ribavirin in patients 70 years of age or older with HCV genotype 1b compensated cirrhosis proved as effective, safe, and well tolerated, as it did in younger patients.

## 1 Introduction

Chronic hepatitis C virus (HCV) infection affects approximately 150 million people worldwide and is the leading cause of cirrhosis and hepatocellular carcinoma when left untreated.^[1]^ Among the genotypes of HCV infection, genotype 1 is the most common, accounting for 60% to 70% of all infections, while subgenotype 1b is predominant in some parts of Europe.^[2]^ It is well-known that in the era of interferon-based therapy, HCV genotype 1 infection was “difficult-to-treat,” as these patients had sustain virologic response (SVR) rates of just 40%.^[3]^

The elderly population is most likely to be infected with HCV and has advanced liver disease as compared to the younger people.^[4]^ Advanced age has been a major limitation of pegylated interferon and ribavirin therapy for chronic HCV infection because of its poor response and tolerability. Consequently, the great majority of elderly patients (if not all, in some countries), defined as those aged 65 years or older, were denied antiviral treatment solely on the basis of their advanced age.^[5]^ In consequence, there is nowadays a large cohort of elderly patients with chronic HCV infection untreated (with interferon-based therapy) and in great need for a new treatment.

Interferon-free regimens are safe and highly effective, allowing treatment for elderly chronic HCV-infected patients without any age limit.^[6–9]^ However, pivotal trials of all oral combinations with direct-acting antivirals (DAAs) included few elderly patients with compensated cirrhosis.^[10–12]^ Twelve-week treatment of HCV genotype 1 compensated cirrhosis with paritaprevir/ritonavir, ombitasvir, and dasabuvir (PrOD) with ribavirin was approved in many countries, including Romania, based on the results of a phase III trial showing an SVR response 12 weeks after the end of therapy (sustained virologic response 12 weeks after the end of treatment [SVR12]) well above 90%.^[13]^ More recently, the HCV regimen of 12-week PrOD without ribavirin reported 100% SVR12 in HCV genotype 1b-infected patients with compensated cirrhosis, meaning that ribavirin does not provide evidence of improving the effectiveness in such patients treated with PrOD.^[14]^

This study aims to assess the real-world efficacy and safety of PrOD with ribavirin for the treatment of HCV genotype 1b compensated cirrhosis in patients aged 70 years and older.

## 2 Methods

### 2.1 Patients

One thousand and eight patients with HCV genotype 1b compensated cirrhosis, treatment-experienced or naïve, were prospectively followed and treated with PrOD + ribavirin for 12 weeks across 10 academic centers of gastroenterology/infectious diseases from all over Romania, between December 1, 2015 and July 31, 2016. Eligible patients were enrolled and assessed following the criteria established by the Romanian National Health Insurance House: adults 18 years of age and above with HCV genotype 1, Child–Pugh class A compensated cirrhosis defined as F4 by Fibromax Biopredictive (Fibrotest score ≥0.75). Exclusion criteria were: decompensated liver cirrhosis, severe chronic kidney disease, documented malignant neoplastic disease, active alcohol consumption, and human immunodeficiency virus coinfection.

All eligible patients signed an informed consent and received treatment with PrOD + ribavirin according to the therapeutic protocol. The PrOD regimen contains paritaprevir 75 mg boosted with ritonavir 50 mg and ombitasvir 12.5 mg (Viekirax, AbbVie Deutschland Gmbh & Co Ludwigshafen, Germany) 2 tablets in a single daily dose, and dasabuvir (Exviera 250 mg AbbVie Deutschland GmbH & Co Ludwigshafen) twice-daily administration. The dose of ribavirin was 1000 mg/day in patients weighting <75 kg or 1200 mg/day in those weighting >75 kg.

This study was approved by National Ethics Committee, and written informed consent was obtained from each patient in accordance with the principles of the Declaration of Helsinki.

### 2.2 Methods

Blood and urine samples were taken for laboratory analyses at baseline, on weeks 4, 8, 12 (end of treatment [EOT]), 12 weeks after the treatment, and whenever it was necessary. Baseline clinical data referred to gender, age, treatment history, comorbidities, and concomitant medication. Laboratory data included HCV RNA level (at baseline, EOT, and SVR12), genotype and subgenotype, liver function tests (aspartate and alanine aminotransferases, bilirubin, alkaline phosphatase, gamaglutamyl transpeptidase, albumin, and international normalized ratio), serum creatinine and creatinine clearance, hemoglobin, platelet count, and alpha-fetoprotein. Child–Pugh and Model of End-Stage Liver Disease scores were calculated at baseline and 12 weeks after the end of therapy. Serum HCV RNA levels were measured with the COBAS TaqMan HCV Quantitative Test (Roche Molecular Systems, Inc. Branchburg, NJ) with a lower limit of quantification and detection of 15 IU/mL.

Efficacy of therapy was assessed by the percentage of patients achieving SVR12 (defined as HCV RNA below the limit of detection 12 weeks after the end of therapy) calculated based on intention-to-treat (ITT) and per-protocol (PP) analysis. ITT population was defined as all patients receiving at least 1 dose of medication while PP population included all patients who completed the 12 weeks of therapy. Safety and tolerability assessment included physical examinations, laboratory data analysis, and all adverse effects (AEs) recorded from the time of the 1st dose of treatment to the last one. Severe adverse events (SAEs), therapy discontinuation, and death rate were recorded.

### 2.3 Statistical analysis

Continuous variables with normal distribution were expressed as mean ± SD, while categorical variables were expressed as absolute values and percentages. The Chi-square test was used to compare categorical data. Quantitative variables with normal distribution were compared using the Student *t* test. For nonnormal data, we used nonparametric methods such as the Mann–Whitney *U* test, while the Kolmogorov–Smirnov test was used to check the normality of the data distributions. The efficacy analysis examined data concerning the total patient population by age at baseline (≥70 or <70 years), whereas the safety analysis described the number and percent of patients with adverse effects or laboratory abnormalities. *P* value less than 0.05 was considered statistically significant. Statistical analysis was carried out using the SPSS 19.0 software (SPSS Inc., Chicago, IL).

## 3 Results

### 3.1 Baseline characteristics

Among the 1008 patients included in our analysis (51.7% females), mean age 59.2 ± 8.7 years (range 33–82), and 117 (11.6%) were aged ≥70 years. Most of the elderly patients were females (58.9%), mean age 73.3 ± 2.8 years (range 70–82), and 37 of them (31.6%) were treatment-experienced. Comorbidities were reported in 60.6% of patients aged ≥70 years compared to 39.8% of those below 70 years (*P* < 0.001). The most frequently met comorbidity in the patients ≥70 years was cardiovascular disease (hypertension, ischemic heart disease, and atrial fibrillation) (Table 1). At baseline, a significant number of patients aged ≥70 years had reduced estimated glomerular filtration rate and hemoglobin level than those <70 years (Table 1). Improvement in the laboratory results was noted at the EOT, while aspartate aminotransferase and alanine aminotransferase values in both age groups were normalized in most of the patients at 4 weeks of therapy. There were no differences in Child–Pugh and Model of End-Stage Liver Disease scores between patients ≥70 and those <70 years of age.

**Table 1:**
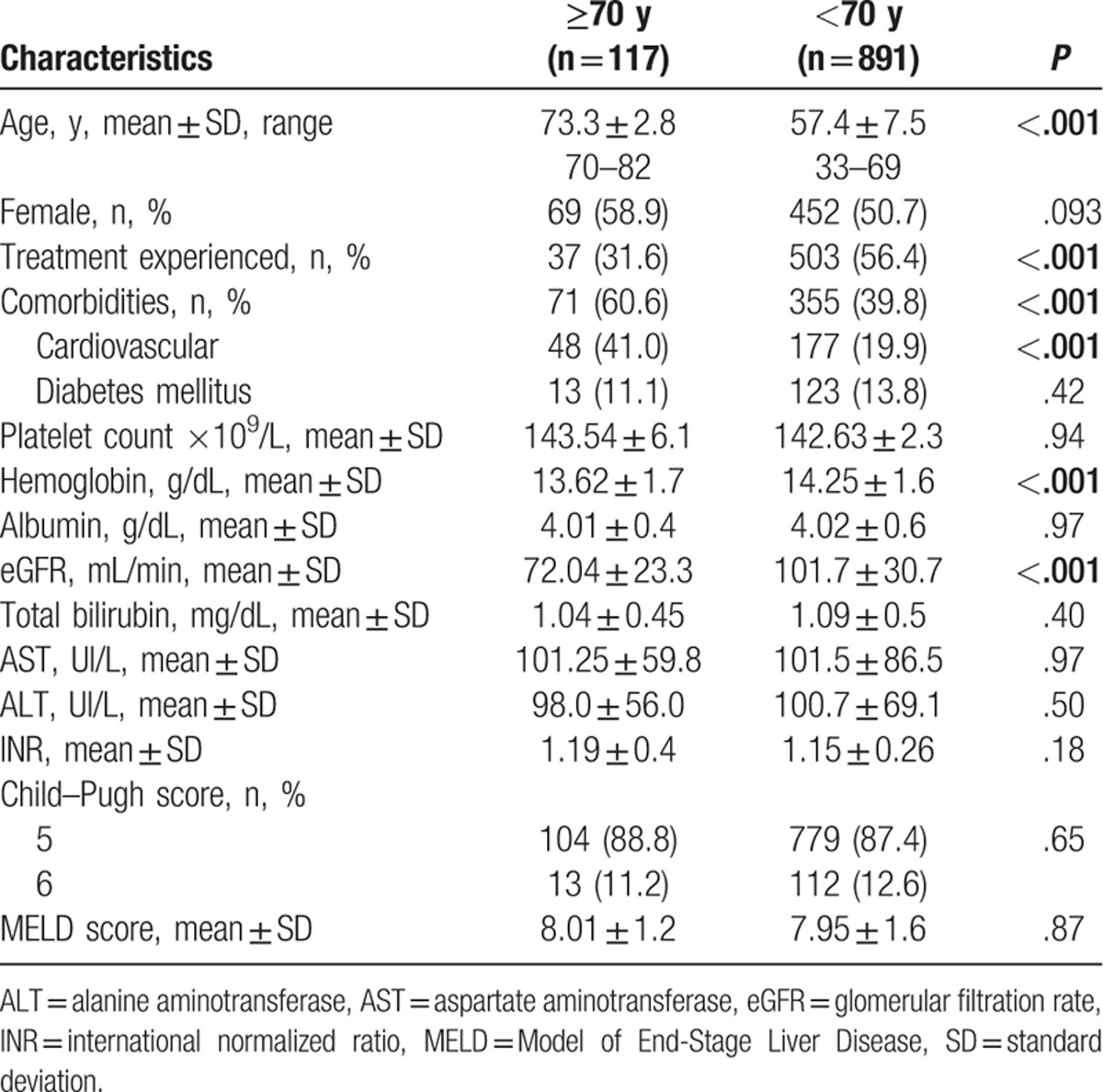
Baseline demographics and laboratory characteristics in patients aged ≥70 and <70 years treated with paritaprevir/ritonavir, ombitasvir, and dasabuvir + ribavirin.

### 3.2 Efficacy

SVR12 rates based on ITT analysis were 97.4% in patients ≥70 years, compared to 97.8% in those <70 years of age (*P* = .82), while SVR12 rates based on PP were 100% in the older group compared to 99.6% in the younger group (*P* = .61), as shown in Table 2. The SVR12 in treatment-naïve patients was 97.5% (78/80) for those ≥70 years of age and 98.2% (381/388) for those <70 years, while for treatment-experienced patients the SVR12 was 97.0% (36/37) for those ≥70 years and 99.4% for those <70 years of age, the differences not being statistically significant.

**Table 2:**
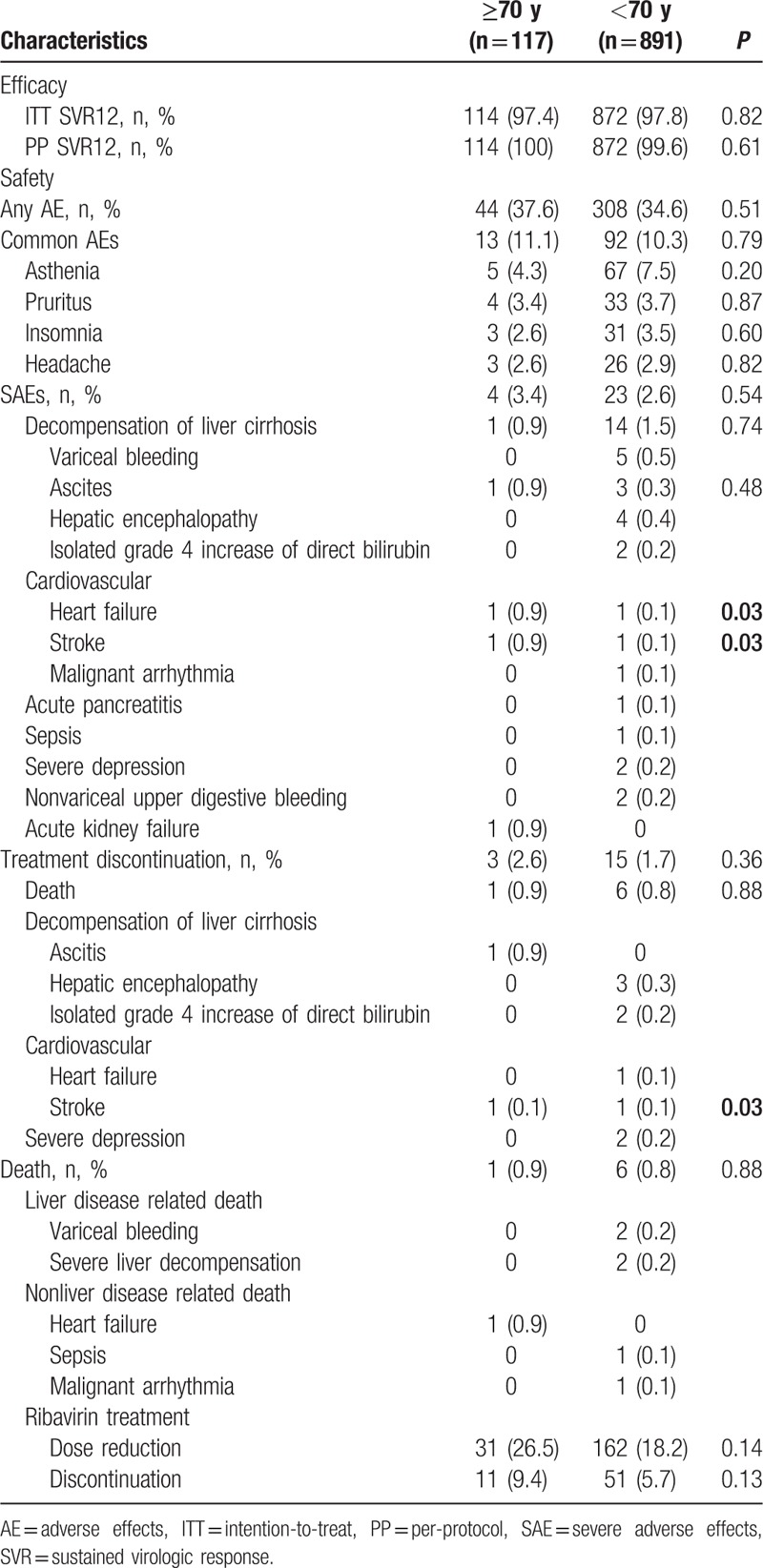
Efficacy and safety of paritaprevir/ritonavir, ombitasvir, and dasabuvir + ribavirin treatment by age.

### 3.3 Safety

A total of 37.6% of patients aged ≥70 years and 34.6% of those <70 years of age (*P* = .51) reported at least 1 AE considered by their physicians as treatment-related (Table 2). The great majority of AEs were mild and manageable, none leading to treatment discontinuation. The most frequent reported AEs in both age groups were: asthenia, pruritus, insomnia, and headache (Table 2). Severe AEs were reported in 4 patients (3.4%) aged ≥70 years (1 decompensation of liver cirrhosis, 1 heart failure, 1 stroke, and 1 acute kidney failure), compared to 23 patients (2.6%) in the group <70 years of age (*P* = .54) (14 decompensation of liver cirrhosis: 5 variceal bleeding, 3 ascites, 4 hepatic encephalopathy, 2 isolated grade 4 increase of direct bilirubin).

One death occurred (0.9%) in a patient aged 79 years (heart failure, not related in any way to PrOD/RBV therapy), and 6 deaths were reported (0.7%) in those under 70 years (2 variceal bleeding, 2 severe liver decompensation, 1 sepsis, and 1 malignant arrhythmia) (*P* = .88) (Table 2). In the elderly group, of the 4 patients with SAEs, 3 discontinued therapy (1 death, 1 liver decompensation, and 1 stroke), and the 1 with acute kidney failure continued therapy after withdrawal of ribavirin. In the younger group, among the 23 patients with SAEs, 15 of them discontinued therapy (6 deaths, 3 hepatic encephalopathy, 2 isolated grade 4 increase of direct bilirubin, 2 severe depression, 1 stroke, and 1 heart failure). Modification of the ribavirin dose (due to anemia and/or increased bilirubin levels) was required in 31 (23.1%) of the patients aged ≥70 years and in 162 (18.2%) of those <70 years (*P* = .14).

## 4 Discussion

The elderly patients with chronic HCV infection, defined in most studies as those aged 65 years or older, were usually denied previous pegylated interferon and ribavirin therapy because of severe adverse effects and poor response.^[5,15]^ Therefore, there is a large cohort in real clinical practice setting of untreated elderly patients with chronic HCV infection and with advanced liver disease. This cohort is in great need for a treatment due to the progressive nature of their disease. Fortunately, interferon-free HCV therapy with DAAs is highly effective and safe, allowing treatment for elderly patients in whom several studies reported similar SVR rates as those obtained in younger patients.^[6–9]^

Controlled clinical trials with PrOD + ribavirin in patients with chronic HCV genotype 1 infection have reported SVR12 rates ranging from 91.8% to 98.3% in cirrhotic and noncirrhotic patients,^[10,11,13,16]^ while with PrOD without ribavirin in HCV genotype 1b noncirrhotic patients SVR12 rates varied from 96.7% to 99.5%.^[11,13,16]^ Poordad et al^[13]^ in a phase 3 clinical trial of patients with HCV genotype 1 compensated cirrhosis (Child–Pugh class A) treated with PrOD + ribavirin for 12 weeks reported SVR12 rates of 91.8% (98.5% in HCV genotype 1b patients). Based on the results of this study, PrOD + ribavirin for 12 weeks regimen has been recommended for patients with HCV genotype 1 compensated cirrhosis.^[17,18]^ More recently, Feld et al^[14]^ have demonstrated that PrOD regimen without ribavirin for 12 weeks was highly effective (100% SVR 12) and well tolerated in HCV genotype 1b patients with compensated cirrhosis, and now this regimen is recommended by both American Association for the Study of Liver Diseases/Infectious Diseases Society of America and European Association for the Study of the Liver guidelines.^[19,20]^ Eliminating ribavirin from this regimen without reducing efficacy will certainly improve the safety profile.

In our real-world cohort of HCV genotype 1b patients aged 70 years or older with compensated cirrhosis treated with PrOD + ribavirin for 12 weeks, the SVR12 rates based on ITT or PP analyses were 97.4% and 100%, respectively, compared to 97.8% and 99.6%, respectively, in cirrhotic patients aged <70 years, the differences being statistically nonsignificant. Of the patients aged ≥70 years, 37.6% reported at least 1 AE considered as treatment-related, a proportion slightly higher but with no statistical significance compared with patients under 70 years of age (34.6%). Most AEs were mild and none was leading to treatment discontinuation. Also, the percentage of SAEs was not significantly higher in patients aged ≥70 years when compared to those less than 70 years of age (3.4% vs 2.6%; *P* = .54). This safety profile is even better than one might expect, considering that all subjects included in the study were older patients with cirrhosis; the safety profile in our study was undoubtedly better than in other studies.^[9,21]^ Such high SVR 12 rates and good safety profiles obtained in our study may be partially explained by the requirements imposed by our national regulations according to which treatment was conducted only in tertiary centers and under the close monitoring of experienced gastroenterologists and infectious diseases specialists.

Our study was carried out in a real-life setting on a homogeneous elderly population (≥70 years of age) with HCV-genotype 1b compensated cirrhosis only, which is what makes it uniquely interesting among many others of its kind. There are but few published studies regarding efficacy of PrOD ± ribavirin in patients with HCV genotype 1 compensated cirrhosis in real life setting.^[9,21–23]^ Thus, Chamorro-de-Vega et al^[21]^ from Spain evaluated in a prospective study the effectiveness and safety in real clinical practice of PrOD ± ribavirin for 12 weeks in patients with chronic HCV genotype 1 (82% genotype 1b) infection and reported a SVR12 rate of 93.8% in cirrhotic patients and 100% in noncirrhotic patients, while AEs occurred in 91.7% of patients (in mild forms, mostly), although none led to premature discontinuation. Of note, patients’ average age was 60 years. The study of Walker et al^[22]^ assessed real-world effectiveness of 2 therapeutic regimens (PrOD and sofosbuvir/ledipasvir) in patients with HCV genotype 1 infection and reported similar high SVR12 rates in both regimens, consistent with results from registrations trials; however, for PrOD regimens with 100% SVR12 rates, the sample size was very low (n = 15) and included only 1 cirrhotic patient and, therefore, no direct comparison with our study is possible. Another published study assessing real-world effectiveness and safety of PrOD ± ribavirin comes from Poland and reported an SVR12 rate of 98.3% in patients with liver cirrhosis, and a higher rate of AEs (72% of cases) than in our study.^[9]^ From Asia (Hong Kong), Chan et al^[23]^ in a retrospective, real-life study including 41 patients with chronic HCV genotype 1 infection (85% had genotype 1b and 61% had compensated liver cirrhosis), PrOD + ribavirin regimen for 12 weeks achieved 95% SVR12 rate, results comparable to the pivotal studies from the West. Similar results have been reported by other studies which included elderly patients treated with PrOD ± ribavirin or other DAAs regimens.^[6,7,24–32]^ Recently, Conti et al,^[7]^ evaluated the efficacy and safety of some DAA regimens in elderly patients, defined as those over 65 years of age with HCV-related advanced fibrosis/cirrhosis, in a real-life clinical setting, and reported that all DAAs regimens used (including PrOD ± ribavirin) were effective and safe in elderly patients with genotype 1b cirrhosis, with SVR12 of 95%. Ioannou et al^[32]^ also reported high SVR rates in the Veteran Affair National Health System patients with HCV genotype 1 and cirrhosis, either treatment-naïve or experienced, treated with PrOD and ribavirin, similar to that obtained under sofosbuvir-based regimens. Saab et al^[6]^ evaluated four open-label phase 3 clinical trials and reported SVR12 of 94% in patients >65 years with HCV genotype 1 cirrhosis who had received ledipasvir/sofosbuvir for 12 weeks, and this regimen proved safe and tolerable for elderly patients.

To our knowledge, our study represents the largest one yet published on PrOD + ribavirin efficacy and safety in patients aged ≥70 years with HCV-genotype 1b compensated cirrhosis in a real-life setting. This study has some strengths such as being prospective, multicentered and including a large number of homogeneous patients ≥70 years of age with HCV genotype 1b compensated cirrhosis only, treated with PrOD + ribavirin. However, our study has also some limitations, the most important one being the absence of assessment concerning long-term impact of SVR12 on the progression of liver disease in elderly patients.

In conclusion, our results demonstrate that a 12-week regimen of PrOD + ribavirin is highly effective, safe, and well-tolerated treatment for patients aged 70 years or older with HCV-genotype 1b compensated cirrhosis, adding new evidence that advanced age should not be a barrier anymore in treating this growing subgroup of HCV patients.
